# Leucasmus or Vitiligo

**Published:** 1880-09

**Authors:** Chas. W. Earle

**Affiliations:** Professor of Diseases of Children, Woman’s Medical College


ARTICLE II.

LEUCASMUS OR VITILIGO. A Clinical Lecture delivered at the Central Free Dispensary. By CHAS. W. EARLE, M.D., Professor of Diseases of Children, Woman's Medical College. Reported by Dr. Julia N. Moss.

*History.*—During the summer of 1879, among the children who presented themselves at the Central Dispensary was the following, afflicted with a disease which in the records of dermatology seems to be somewhat rare.

Maggie Conlan, aged twelve, born in Chicago, of Irish parentage, resides at 394 Hubbard street, the sanitary condition of the neighborhood being good.

The family history is unmarked. Her maternal grandfather died of consumption, and her father is subject to headache, and pain in side, has a few gray hairs and is thirty-seven years of age.

Patient's health prior to four years ago was good. At that time she had occasional, but not severe headaches; this continued for three years, when headaches appeared daily, about 11 a. m., continuing an hour and returned in the afternoon. During this period she had also complained of irregular pains in the chest. In the winter, 1878–79, she suffered from irregular chills, twice daily, which continued about ten months, appetite poor, bowels constipated. Her headaches, which previously had been intermittent, became constant, and her hair began to turn gray, and the skin of the forehead became very white. White spots appeared upon the neck, and one upon the body, the skin of the face elsewhere became very yellow, some yellow spots also appeared upon the neck and chest.

When first seen in the early summer of 1879, she presented the usual development of that age, well nourished, but slightly anæmic, but complained of great weakness, and had had a hacking cough for some months, voice very hoarse; examination revealed a follicular pharyngitis, but lung sounds were normal, and circulation good.

There were various tender spots in the left axillary region, also in the right infra-clavicular, and in the posterior region of the chest, to the right of the spine, but no headache for past three weeks. An irregular line ran around the forehead, above which the skin was intensely pearly white; there were also two white spots each side of the neck, and one over the lower portion of sternum, skin of face and body yellowish, also two small yellow patches upon the forehead. Hair was fully one-half gray, and growing very low upon forehead.

Patient was put upon a combination of iron, quinine and strychnine.

August 10.—Grayness of hair is disappearing, also the marked yellow and pearly white tints of the skin are becoming obliterated.

September 8.—Appetite better, but dizzy in the morning, headache has returned, bowels regular, but pain in abdomen, hoarseness and cough continue.

Abdominal pain continuing through the month with an attack of diarrhœa (possibly consequent upon a cold), the tonic treatment was discontinued, and a prescription given, containing bismuth, pepsin and acid muriatic dil., which entirely relieved abdominal symptoms.

In the early part of October the patient had intermittent attacks of severe pain in right chest, which were soon relieved by cinchona; continued to improve until December 12th, when visits were discontinued.

May, 1880, patient again presents herself at dispensary, headache has returned, peculiar discoloration of the skin intensified, grayness of hair also increasing, bowels regular, appetite medium; child has become exceedingly irritable in disposition, and very hard to control, giving parents much anxiety and trouble.

The history having been read, the lecturer stated that he had taken the trouble to visit the child and to have a conversation with her mother.

A re-examination of the body was made, and leucasmic spots were found at the ensiform cartilage and investing the left nipple.

Brown spots were also found in different parts of the chest, but none extending below a point corresponding with a line drawn around the body at the umbilicus.

With regard to the irritability of the child, as mentioned in the history, the mother remarked to the Professor that she had often thought, "if the good Lord would take the child away (glory be to His name), she (the mother) would not be *displazed.*"

Patients with this disease do not come to our notice in any considerable number. Prof. Maynard, who has had charge of this class of diseases in this institution for over nine years, and has treated during that time 3000 consecutive skin diseases, has seen three cases. Erastus Wilson speaks of only twelve cases. Duhring says the affection is rare.

There is some reason to suppose that in other countries, India, for instance, it is more common. Tilbury Fox remarks, in regard to its frequency in that country, that the well-to-do natives suffer from it. Nearly all dermatologists, as far as I have had access to their works, agree that it is a disease of pigmentation, accompanied with more or less debility of the nervous powers.

The *synonyms* are numerous, and vary as authorities base their classification upon the presumed *cause* of the disease or the *visual appearance* of the morbid integument.

From among these many names I will only mention leucasmus, achroma, alphosis, albinismus, acquired piebald skin and vitiligo.

It is a malady confined to the retemucosum, and may be *general* or *partial.* If the disease were general, it would usually be congenital, and there would be uniformly an absence of pigment over the whole surface of the body. To this class of people the name of Albino has been applied, concerning whom I do not purpose at this time to make any remarks. Most of you, undoubtedly, have seen these individuals, as they are exhibited throughout the country.

Coming now to speak more particularly of the acquired achromic condition or *partial* leucasmus, we find that it is probably most common among the negro race, occasionally, however, being found among the whites, and usually associated with some form of diseased innervation and nutrition.

In the little patient now before us we find a long history of malaria, neuralgia and anæmia. Indeed it was not until she had suffered with irregular chills and tender spots and attacks of neuralgia for more than two years that she began to notice this peculiarity in her integument.

It will be noticed that the white spots, of which there are several, terminate abruptly, and that the surrounding skin, in some instances at least, is of a darker color than usual—that is we find an absence of pigment in the parts afflicted with leucasmus and an increase of this same formation in the contiguous integument.

Referring almost inadvertently to a *deposition* of pigment in the derma, when the case before us is an absence of the same I may be allowed to speak briefly of the different chromatogenous diseases we find manifesting themselves in the variations of color of the skin.

We find four varieties and they occur, as regards frequency in about the order which I will give them:

Melanoderma or melasma;

Xanthoderma;

Leucoderma;

Cyanoderma.

The first is a deposit of, or change to black pigment and the best types, of course, are found in the colored race. We have however at least one pathological condition in which dark deposits take place; that is Addison's disease, and there is a physiological process during the course of which a partial or local melasma is frequently seen. I refer to the change in color which takes place around the nipple during pregnancy. The best illustration of xanthoma or yellow discoloration is the round flat patch commonly called a freckle, to which, according to some authorities, should be added chloasma or pityriasis versicolor, familiarly known among the people as liver spots. Leucoderma, or white spots, is the subject of this lecture, and cyanoderma or blue discoloration of the skin is so rare that many dermatologists believe it a myth.

In regard to this classification it is proper for me to say that the writers of our standard books on skin diseases differ greatly. Some class all under diseases of the pigment system. Others speak of the xanthoderma as a *new growth* and Vitiligo under the head of *atrophies*. Concerning this difference of authors I can say nothing at this time, but will only attempt to present an epitome for your guidance as general practitioners.

As to the *causes* of leucasmus we can say but little. Deficient innervation has certainly to do with it, as also has depressing diseases, particularly those of malarial origin, when long continued. There is also considerable testimony which goes to show that exposure to the sun's rays, especially in hot climates, may be a producing cause.

The *diagnosis* is not difficult. You will find white spots upon a skin which is not at all changed in structure. Where leucodermic patches take place on the scalp, you frequently find the hair white, as in the case before us. In the leprous affections we have whiteness of the skin, but in addition such a *thickening* and *infiltration*, associated with so many other symptoms of a severe order, that you will hardly have trouble in differentiating from the local disease under consideration.

*Treatment.*—This patient has always improved when placed on tonics. Indeed, after the physician is assured that all the functions of the body are well and properly performed, tonics, perhaps of the alterative varieties, are the medicines indicated. The treatment at best will not be particularly successful. You should, however, pay particular attention to digestion, secretion and excretion, and then give the ferruginous preparations with quinine. The ordinary tr. ferri chloridi with quinine is excellent, or the mineral acids with quinine. Arsenic with iron and some of the varieties of cinchona will be indicated at times. Strychnia will be valuable in some cases, as also will malt and cod liver oil, and syrup of the iodide of iron.

In the treatment of this disease, or any other in which you desire to prescribe quinia and syrup ferri iod., you will find the following combination an excellent one:

| | | |
|---|---|---|
| ℞ | Quiniæ sulph.................................... | 1.0 |
| | Syrupi ferri iodidi................................ | 8.0 |
| | Acidi citrici .......................................... | 0.5 |
| | Glycerinæ............................................. | 8.0 |
| | Aq. anisi q. s. ad.................................. | 50.0 |

M. Give a teaspoonfnl four times a day.

While you are improving the nutrition, and imparting tone and vigor to lowered innervation, do not neglect the application of some good stimulus to the skin. Apply friction, give baths, and use some of the soaps usually recommended in skin diseases, such as the carbolic acid or tar varieties.

We will give the patient before us the advantage of the treatment suggested, and try at some future time to again present her to the class, in order that you may note the result.

